# Unveiling psychobiological correlates in primary Sjögren’s syndrome: a machine learning approach to determinants of disease burden

**DOI:** 10.3389/fpsyt.2025.1549756

**Published:** 2025-06-03

**Authors:** László V. Módis, András Matuz, Zsófia Aradi, Ildikó Fanny Horváth, Antónia Szántó, Antal Bugán

**Affiliations:** ^1^ Department of Behavioural Sciences, Faculty of Medicine, University of Debrecen, Debrecen, Hungary; ^2^ Szabolcs-Szatmár-Bereg County Teaching Hospital, Nagykálló Sántha Kálmán Member Hospital, Nagykálló, Hungary; ^3^ Department of Behavioural Sciences, Medical School, University of Pécs, Pécs, Hungary; ^4^ Szentágothai Research Centre, University of Pécs, Pécs, Hungary; ^5^ Division of Clinical Immunology, Department of Internal Medicine, Faculty of Medicine, University of Debrecen, Debrecen, Hungary

**Keywords:** primary Sjögren’s syndrome, machine learning, biopsychosocial model, autoantibodies, symptom burden, psychobiological correlations, temperament and character, state and trait anxiety

## Abstract

**Introduction:**

Besides primary Sjögren’s syndrome (pSS) is generally assessed through biological markers, growing evidence suggests that psychological and social factors—such as anxiety, depression, personality traits, and social support—may also play a role in disease burden. Relative contribution of these biopsychosocial dimensions to disease activity in pSS, however, has not been quantitatively compared. This study aimed to evaluate the predictive weight of different factors in determining both objective and subjective disease burden using machine learning (ML) models.

**Methods:**

117 pSS patients, whose biological (blood cell counts, complement activity, IgG, RF, SSA, SSB), psychological (personality traits, depression, anxiety, basic self-esteem assessed via self-reported questionnaires), and social (socioeconomic status and social support) measures were collected in a composite database. Outcome variables were SSA/SSB autoantibodies and EULAR Sjögren Syndrome Patient Reported Index (ESSPRI), as indicators of biological and perceived disease burden, respectively. Three machine learning algorithms were trained to predict outcome variables, first by each measure category, then on the entire set of predictor variables. Permutation feature importance was used to assess the importance of the predictors. The five most important predictors were selected for all target outcomes.

**Results:**

Concerning autoantibodies, the model performed best with biological input only, in the case of ESSPRI, the complete dataset gave the best performance. Trait anxiety was selected as important negative predictor of both autoantibodies. Besides, biological measures (IgG, RF, platelet count) and age were among the five most important features. State anxiety and temperament trait ‘Fatigability’ were important positive predictors of ESSPRI, while character trait ‘Pure-hearted conscience’, IgG and RF were important negative predictors.

**Conclusions:**

Unexpected psychobiological correlations, like trait anxiety and IgG/RF as negative predictors of autoantibodies and ESSPRI, respectively, suggest different (immunobiological and psychosomatic) disease mechanisms and symptom burden. Importance of psychological factors in estimating disease burden may pave the way toward novel, more sensitive diagnostic tools and therapeutic methods and better understanding of pathomechanisms of pSS.

## 1 Introduction

Primary Sjögren syndrome (pSS) is a chronic autoimmune exocrinopathy, which main clinical hallmarks are dryness of the mouth (xerostomia) and the eye (xerophthalmia, keratoconjunctivitis sicca), fatigue and joint pain. Besides, several extraglandular manifestations may occur, affecting inter alia the central and peripheral nervous system and the mental health as well (1). PSS has a pronounced female propensity and is more prevalent in the Caucasian population. The mean age of onset is usually in the 40s to 50s (2). A recent study draws the attention to the increasing incidence and prevalence of autoimmune diseases worldwide (by 19.1% and 12.5% yearly, respectively), and yet the understanding of these conditions is still fragmentary (3).

In line with the biopsychosocial model of health, pSS, as a chronic disorder is evoked and regulated by multiple factors. As for the biological side, wide range of autoantibodies can be detected in pSS, associated with the autoimmune inflammation, among which anti-Ro/SSA (SSA), anti-La/SSB (SSB) and rheumatoid factor (RF) are the most common ones, but cryoglobulins and antinuclear antibodies may also be produced. Besides being a diagnostic sign of pSS, the presence of SSA and SSB (detected in 60-70% of the patients) is associated with earlier disease onset, sicca symptoms and extraglandular manifestations together with other B-cell activation markers (4).

Psychological factors, such as depression, anxiety, cognitive symptoms and many more have long been known as phenomena determining the mental health of pSS patients (1, 5–7). Personality traits typical for pSS have also been identified, thus, hypochondria, depression, hysteria domains of the Minnesota Multiphasic Personality Inventory (1, 8), neuroticism and psychoticism scales of the Eysenck Personality Questionnaire (9) and neuroticism scale of the Revised NEO Personality Inventory (10) were proven to be more pronounced among these patients. Milic et al. found lower levels of Extraversion and Openness domain of the NEO-PI-R among pSS patients compared to healthy controls. Furthermore, these personality traits were associated with the severity of dryness, pain and fatigue administered by ESSPRI, indicating that personality traits and perceived symptom severity are related to each other (11). The psychobiological model of personality, which would allow to establish neurobiological correlations of personality traits, however, has not been investigated in pSS to our knowledge. Very few information is available concerning the disease modifying effect of social factors regarding this condition, although its characteristics (female predominance, high geographical and cultural variation, late onset) may indicate socioeconomic impact besides the genetical one.

As for the methods, there is increasing interest around artificial intelligence and machine learning (ML) in science generally and in medical research specifically. So far, studies conducted using ML in pSS aimed the technological improvement of diagnostic tools (12–14). Factors involved in the development and modification of the disease have not been studied this way. The disease is highly heterogenous in its manifestation, making it difficult to reveal, how different mechanisms influence it in the background. Furthermore, most prior studies have examined different domains in isolation, limiting the understanding of how psychological and social characteristics compare in predictive strength to traditional biological markers. Hence, the study aim was to estimate the relative importance of biological, psychological, and social factors in predicting both objective immunological markers (SSA/SSB autoantibody status) and subjective disease burden (ESSPRI scores) in pSS. Given the high-dimensional dataset involving many diverse variables, ML algorithms were preferred over traditional biostatistical methods due to their ability to handle complex data with many correlated variables and to potentially reveal nonlinear relationships (15).

The hypotheses of the study were the followings: [1] In the case of autoantibodies, it was expected that their serum levels would be primarily predicted by biological factors, with psychological and social factors also contributing to a lesser extent. [2] Regarding ESSPRI, it was speculated that psychological and social variables would serve as more important predictors than biological ones. [3] According to previous assumptions, impaired psychological functions (e.g. higher levels of depression or anxiety) will predict worse immunological status and higher disease activity scores. [4] Finally, it was hypothesized that among temperament and character traits, Harm Avoidance would exert the most significant influence on the prediction of subjective disease burden, given its previously reported associations with neuroticism (16) - a personality dimension found relevant in pSS, as mentioned above- as well as its links to serotonergic functioning and hence, affective symptoms.

## 2 Materials and methods

### 2.1 Materials

#### 2.1.1 Participants

The data was collected at the Autoimmune Sjögren specialty clinic, Division of Clinical Immunology, Institute of Internal Medicine, Clinical Centre, University of Debrecen. The initial dataset consisted of 127 patients, 10 participants were excluded later due to incomplete information, resulting finally in 117 patients (105 females, 12 males; aged between 30 and 82 years, mean age = 59.62 ± 13.22). The inclusion criteria were the primary nature of the disease and intact cognitive functions. The study was conducted according to the guidelines of the Declaration of Helsinki and approved by the Institutional Ethics Committee of the University of Debrecen (protocol code: 5614-2020, date of approval: 17.12.2020). Informed consent was obtained from all subjects involved in the study.

### 2.2 Measures

#### 2.2.1 Sociodemographic questions

The patients answered a list of sociodemographic questions covering various background information including gender, age, highest level of education, settlement type, smoking habits, family status, number of children and satisfaction with family life.

#### 2.2.2 Biological measures

First, laboratory data of the patients including neutrophil granulocyte count (Neu), lymphocyte count (Ly), hemoglobin concentration (HGB), platelet count (PLT), complement component C3, C4 and total complement activity (C3, C4 and CH50 respectively), and serum levels of immunoglobulin G (IgG), rheumatoid factor (RF), anti-Ro/SSA (SSA) and anti-La/SSB (SSB) autoantibodies were collected.

#### 2.2.3 Psychological measures

Subsequently, questionnaires and inventories were registered. Revised Temperament and Character Inventory (TCI-R) was applied to assess personality traits. This inventory was chosen because it allows to establish differences between inherited and acquired domains of personality, making possible more delicate conclusions regarding the pathomechanism therefore. The inventory consists of seven scales. The first four are temperament scales, which is considered innate, individual pattern of associative learning that are highly genetically determined and correlate with different neurotransmitters, in the terms of novelty, danger and punishment and reward, and further three are character scales, which are indicators of the maturity of personality and are related to acceptance of the individual self, acceptance of other people, and acceptance of nature in general (17–19). Higher scores on the subscales indicate a more pronounced trait. All scales and subscales were evaluated separately.

Besides, depression was measured applying a shortened, 9-item version of the Beck Depression Inventory (BDI) (20–22). To appraise anxiety, 40-items State-Trait Anxiety Inventory (STAI) was administered (23, 24). Higher scores on these inventories reflect greater depression/anxiety respectively. 18-item version of Basic Self-Esteem Scale (BSE) was also involved, where higher scores denoted stronger self-esteem traits (25, 26). To examine social support available for the patients, 20-item Medical Outcomes Study Social Support Survey (MOS-SSS) was used, in which higher scores on the scales expressed better terms of social support (27–29). The questionnaires were chosen based on their clinical relevance measuring the required variables and considering the compliance of the patients. In the case of all questionnaires and inventories, the previously validated Hungarian version was applied, except for BSE, where only preliminary examinations were conducted (see references above). The questions could be answered on Likert scales (0–3 points in BDI, 1–4 points in STAI, 1–5 points in TCI, BSE and MOS SSS). The detailed structure of the scales and facets of the applied questionnaires are shown in **Table 1**.

**Table 1:** Cronbach’s alpha values of the scales applied in the investigation.

Scale	SD	Cr.α
Temperament and Character Inventory		
Novelty seeking (NS)	0.283	0.720
*Exploratory excitability (NS1)*	*0.485*	*0.661*
*Impulsiveness (NS2)*	*0.395*	*0.502*
*Extravagance (NS3)*	*0.444*	*0.616*
*Disorderliness (NS4)*	*0.452*	*0.498*
Harm avoidance (HA)	0.430	0.863
*Anticipatory worry (HA1)*	*0.489*	*0.725*
*Fear of uncertainty (HA2)*	*0.547*	*0.615*
*Shyness (HA3)*	*0.511*	*0.523*
*Fatigability (HA4)*	*0.648*	*0.766*
Reward dependence (RD)	0.369	0.796
*Sentimentality (RD1)*	*0.465*	*0.511*
*Openness to warm communication (RD2)*	*0.470*	*0.666*
*Attachment (RD3)*	*0.661*	*0.719*
*Dependence on approval by others (RD4)*	*0.561*	*0.573*
Persistence (PS)	0.386	0.864
*Eagerness of effort (PS1)*	*0.493*	*0.707*
*Work hardened (PS2)*	*0.468*	*0.623*
*Ambitious (PS3)*	*0.406*	*0.566*
*Perfectionist (PS4)*	*0.502*	*0.611*
Self-directedness (SD)	0.332	0.832
*Responsibility (SD1)*	*0.536*	*0.688*
*Purposefulness (SD2)*	*0.571*	*0.611*
*Resourcefulness (SD3)*	*0.506*	*0.537*
*Self-acceptance(SD4)*	*0.546*	*0.758*
*Enlightened second nature (SD5)*	*0.382*	*0.598*
Cooperativeness (C)	0.331	0.816
*Social acceptance (C1)*	*0.435*	*0.600*
*Empathy (C2)*	*0.507*	*0.511*
*Helpfulness (C3)*	*0.419*	*0.481*
*Compassion (C4)*	*0.588*	*0.744*
*Pure-hearted conscience (C5)*	*0.446*	*0.512*
Self-transcendence	0.475	0.834
*Self-forgetfulness (ST1)*	*0.582*	*0.704*
*Transpersonal identification (ST2)*	*0.624*	*0.765*
*Spiritual acceptance (ST3)*	*0.681*	*0.747*
Basic self-esteem (BSE)- libido	0.417	0.632
Basic self-esteem (BSE) - aggression	0.603	0.560
Beck Depression Inventory	0.561	0.894
State anxiety	0.576	0.934
Trait anxiety	0.533	0.897
MOS-SSS Emotional/informational support	0.752	0.919
MOS-SSS Tangible support	0.858	0.875
MOS-SSS Affection	0.880	0.865
MOS-SSS Positive social interaction	0.844	0.820

SD, standard deviation; Cr. α, Cronbach’s alpha; MOS-SSS, Medical Outcomes Study Social Support Survey.

#### 2.2.4 Disease burden

Since disease activity is the outcome variable in the applied statistical model, defining it is an important point in this study. First, perceived disease activity was detected, based on subjective judgment of the patients. Therefore, participants completed the EULAR Sjögren’s Syndrome Patient Reported Index (ESSPRI), which first three questions measure the three hallmark symptoms of the disease (dryness, pain, fatigue) on a 10-point numeric scale, which average gives the ESSPRI score (30). The other target outcomes were SSA and SSB autoantibodies. Since SSA and SSB are well known to be associated with more pronounced extraglandular symptoms and overall less favorable disease course (see reference in Introduction), these antibodies were used as a variable of objective disease burden.

### 2.3 Statistical analyses

All programming was implemented in Python, using the scikit-learn (version = 1.0.2.) package (31). The dataset was split into training (80%) and test datasets (20%). To avoid information leakage, feature selection as well as parameter tuning were conducted on the training set only. Prior to model training, data were standardized using Z-transformation. Feature selection was carried out using the Least Absolute Shrinkage and Selection Operator (LASSO) with 10-fold cross-validation. Feature importances were obtained by permutation feature importance (n = 100).

For the prediction of target outcomes (i.e. ESSPRI score, SSA and SSB), three algorithms were used: LASSO regression, elastic net regression and support vector regression (SVR). These algorithms were selected for several reasons. First, these methods are widely used and thus, their use may enhance potential comparisons with other studies focusing on autoimmune diseases (e.g (32–34). Second, elastic net regression was selected because of its capability of handling correlated predictors (e.g. laboratory data, TCI-R subscales), while LASSO regression was selected because sparse models were preferred to enhance generalizability by preventing overfitting. In addition, both of these linear algorithms have the advantage of providing rather interpretable models. Finally, SVR was selected to potentially capture non-linear associations and complex relationships enabled by SVR’s kernel trick.

For LASSO and elastic net regression, the parameter alpha was tuned (number of alphas = 500). For SVR, the parameters C {10^0^, 10^1^, 10^2^} and gamma {10^-2^, 10^-1^, 10^0^} were tuned with either radial basis function or linear kernel. Model performance was assessed by R^2^, and root mean square error (RMSE). Model training was achieved by 10-fold cross-validation. The final model evaluation was based on predictive performance achieved on the previously unseen testing dataset. To account for random fluctuations due to random data splitting, the whole procedure was repeated 100 times with different randomization seeds [following Matuz et al. (35)]. Since the distribution of performance metrics was found to be skewed, median was used to describe the center of the distribution and the interquartile range to describe the dispersion of the distribution.

For each outcome variable, the analyses were run with four different sets of predictor variables. First, only biological variables were entered into the model. Second, only psychological variables were entered, while third, only socio-demographic variables were entered into the model. Fourth and final, all three types of variables were used for model training. The aim of this procedure was to directly compare the predictive power of the three types of variables and to compare the complex models (i.e. the ones trained on many kinds of variables) with the sparser models (i.e. the ones trained on one kind of variables).

## 3 Results

### 3.1 Clinical data and questionnaires

The Cronbach’s α values of the questionnaires are shown in **Table 1**. The 7 TCI traits were proven to be reliable (Cronbach α values between 0.72 and 0.86), while their facets showed weaker internal consistency (α =0.48-0.77). Subscales for BSE libido (α =0.63) and aggression (α=0.56) were also less reliable, BDI, STAI and MOS-SSS on the contrary were proven to be reliable scales (α =0.82-0.93). **Table 2** demonstrates descriptives of the features later selected most frequently in the statistical model.

**Table 2:** Descriptive statistics of the three outcome variables and the top features.

Variable	Mean	SD	CI95%
Outcomes			
ESSPRI score	5.32	2.20	4.92 - 5.72
SSA	43.29	34.62	37.02 - 49.56
SSB	30.63	32.89	24.67 - 36.59
Top predictors			
Age	59.62	12.08	57.43 - 61.81
Fatigability (TCI HA4)	25.84	5.22	24.89 - 26.79
Pure-hearted conscience (TCI C5)	31.16	3.55	30.52 - 31.80
State Anxiety	41.78	11.57	39.68 - 43.88
Trait anxiety	44.31	10.45	42.42 - 46.20
Hemoglobin concentration	135.94	12.90	133.60 - 138.28
Immunoglobulin G	14.28	7.81	12.86 - 15.70
Rheumatoid factor	34.39	85.79	18.84 - 49.94
Platelet count	230.89	50.58	221.72 - 240.06

SD, standard deviation; CI95%, 95% confidence interval; ESSPRI, EULAR Sjögren’s Syndrome Patient Reported Index; SSA, anti-Ro/SSA autoantibody; SSB, anti-La/SSB autoantibody; TCI, Temperament and Character Inventory; HA, Harm avoidance; C, Cooperativeness .

### 3.2 Feature selection

**Figure 1:**
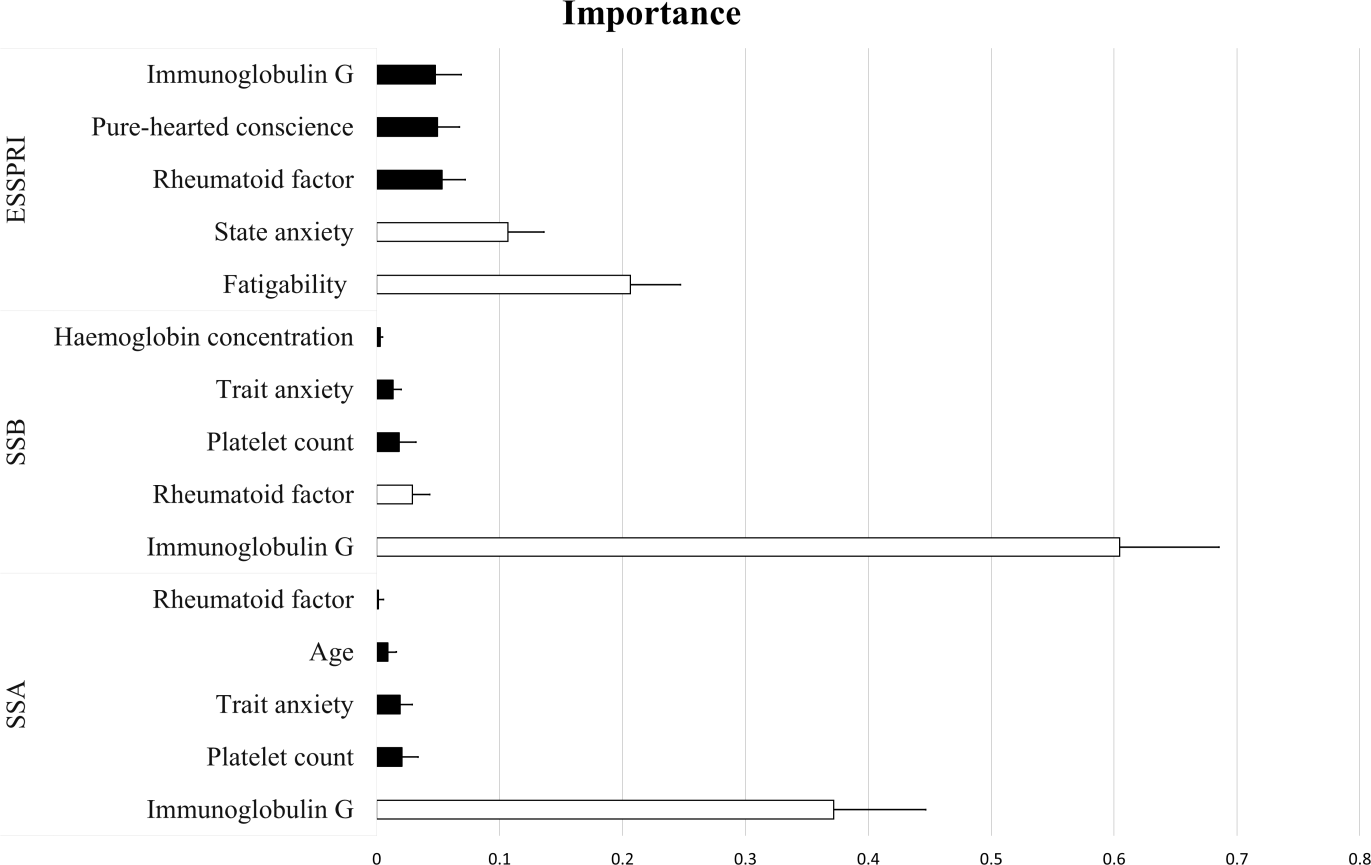
Importance values of the most frequently selected features based on LASSO regression. White and black colors indicate positive and negative predictors, respectively. Error bars represent the standard deviation. Fatigability represents the 4^th^ facet of Harm avoidance trait, while Pure-hearted conscience represents the 5^th^ facet of Cooperativeness trait of the Temperament and Character Inventory. SSA, anti-Ro/SSA antibody; SSB, anti-La/SSB antibody; ESSPRI, EULAR Patient Reported Index.

**Figure 1** depicts the importance values of the most important features. Biological variables, immunoglobulin G and rheumatoid factor were among the most important features in all three outcomes. They were positively associated with SSA and SSB, while negatively associated with ESSPRI scores. Platelet count was also one of the most important predictors of SSA and SSB. Higher levels of platelet count were associated with lower levels of both SSA and SSB. In addition, hemoglobin concentration was also one of the top predictors of SSB: higher hemoglobin concentration was associated with lower SSB.

Among the psychological variables, trait anxiety was one of the most important predictors of both SSA and SSB, showing a negative relationship with both outcomes. In contrast, for the prediction of ESSPRI scores, the other anxiety-related variable, state anxiety, was selected: higher state anxiety was associated with higher ESSPRI scores. For the prediction of ESSPRI scores, two TCI factors, Fatigability and Pure-hearted conscience, were also among the top predictors. In fact, Fatigability was the most important predictor of ESSPRI scores, and the analyses showed a positive relationship between the two, while Pure-hearted conscience was negatively related to ESSPRI scores. A sociodemographic variable, age, was also found to be important in case of SSA and ESSPRI: older age was associated with lower levels of SSA and higher levels of ESSPRI (mean importance = 0.03, ß = 0.02), however, in the latter case age was not among the top five predictors. Beside age, the top 10 predictors of ESSPRI also included BSE libido (mean importance = 0.03, ß = 0.05), Extravagance (TCI NS3) (mean importance = 0.03, ß = -0.05), hemoglobin concentration (mean importance = 0.03, ß = -0.02) and BDI (mean importance = 0.03, ß = 0.05).

### 3.3 Model performance

The results of ML algorithms are summarized in **Table 3**. Here, only the most important findings are reported. For the prediction of ESSPRI scores, the best predictive performance was achieved by the SVR when it was trained on all three kinds of measures. However, a similar performance could be reached by the elastic net regression as well, when trained on psychological variables only. For the prediction of SSA and SSB, the models performed best when they were trained on biological measures only. In case of both outcomes, the best predictive performance was achieved by the LASSO regression. The LASSO regression-based prediction of SSB yielded the overall best performance with an R^2^ of 0.44 when trained on biological data only.

**Table 3:** Predictive performances of the machine learning algorithms.

Features\ Algorithms	Outcomes					
	ESSPRI		SSA		SSB	
	*R^2^*	*RMSE*	*R^2^*	*RMSE*	*R^2^*	*RMSE*
BIO						
Elastic net	.08 (.21)	2.05 (.28)	.23 (.18)	29.64 (4.26)	.42 (.16)	24.47 (3.97)
LASSO	.06 (.21)	2.06 (.25)	.24 (.21)	29.77 (4.29)	.44 (.16)	23.73 (3.62)
SVR	.09 (.20)	2.09 (.28)	.16 (.30)	31.64 (5.75)	.28 (.33)	26.25 (7.59)
PSY						
Elastic net	.14 (.37)	2.01 (.39)	-.14 (.20)	36.51 (3.37)	-.18 (.24)	34.70 (5.63)
LASSO	.12 (.39)	2.02 (.38)	-.19 (.23)	36.99 (4.30)	-.23 (.32)	35.79 (5.97)
SVR	.09 (.32)	2.04 (.37)	-.37 (.38)	39.28 (5.46)	-.43 (.28)	38.07 (8.46)
SOC						
Elastic net	.03 (.27)	2.08 (.38)	-.06 (.17)	34.97 (3.64)	-.07 (.19)	33.59 (5.98)
LASSO	.03 (.27)	2.09 (.40)	-.09 (.22)	35.56 (4.18)	-.12 (.23)	33.86 (6.53)
SVR	.03 (.19)	2.12 (.38)	-.19 (.31)	37.18 (5.16)	-.33 (.27)	36.70 (8.01)
BIO+PSY +SOC						
Elastic net	.15 (.35)	1.98 (.37)	.14 (.27)	31.04 (5.56)	.35 (.20)	25.74 (4.61)
LASSO	.14 (.36)	1.99 (.39)	.14 (.29)	31.09 (5.81)	.36 (.25)	25.79 (5.01)
SVR	.17 (.26)	1.99 (.42)	.04 (.42)	33.26 (6.25)	.33 (.31)	26.62 (6.99)

BIO, biological features; ESSPRI, EULAR Sjögren’s Syndrome Patient Reported Index; SSA, anti-Ro/SSA autoantibody; SSB, anti-La/SSB autoantibody; PSY, psychological features; RMSE, root mean squared error; SOC, sociodemographic features; LASSO, Least Absolute Shrinkage and Selection Operator SVR, support vector regression. Evaluation metrics represent the median values over 100 iterations. Numbers in the paratheses represent the interquartile range.

## 4 Discussion

To our knowledge, this is the first study investigating the strength of the effect of biological, psychological and social health determinants on disease activity in pSS in one model, using ML. As the results indicate, considering SSA and SSB, the best predictive performances were achieved when using biological models only. In the case of the subjective disease activity marker ESSPRI, the best performance was observed when all disease modifying clusters (i.e., biological, psychological and social factors) were entered into the model simultaneously. Although many papers described the correlation between particular biological, psychological, social factors and disease activity, no comprehensive study was performed to analyze the weight and proportion of their importance in disease modification.

Concerning the main findings, for the prediction of SSA and SSB, the best predictive performance was achieved using biological measures alone. Trait anxiety was the only psychological variable selected as a negative important predictor for both autoantibodies. Besides, biological factors were selected by the model. The proportion of the selected important predictors is congruent with hypothesis 1. Immunoglobulin G count turned out to be the most important predictor of both SSA and SSB, since the majority of these molecules belong to the IgG type (36). Rheumatoid factor, as another common autoantibody present in pSS, was also selected among the five most important predictors for both autoantibodies. Platelet count was the second most important predictor in the case of SSA and the third one in SSB, showing negative associations.

In the case of ESSPRI, combining biological, psychological, and social factors improved model performance, consistently with hypothesis 2. As for the outcomes, Fatigability subscale of the Harm avoidance personality trait turned out to be the most important direct predictor, followed by state anxiety. Pure-hearted conscience (C5) facet of the Cooperativeness trait, however, was proven to be an important negative predictor, alongside with RF and IgG. Hypothesis 4 stated that Harm avoidance was expected to be an important prior to the investigation. This assumption was partially justified, since one of its subscales was uncovered as the most important predictor. Pure-hearted conscience (C5) being an important predictor, however, was an unexpected result. Contrary to the premises stated in hypothesis 1 and 2, social factors and demographic variables, except for age, showed negligible, if any influence on the target outcomes.

Depression and anxiety have long been known as symptoms of pSS (1, 5, 8–10, 37), however, their association with autoantibody production received little empirical support yet. The revealed negative association between trait anxiety and autoantibodies contradicts hypothesis 3, since trait anxiety, which causes greater psychological burden, predicts lesser objective disease burden. One study reported that pSS patients without SSA antibodies experienced a greater psychological burden (9), which is congruent with the present finding of a negative association between autoantibodies and trait anxiety, since trait anxiety has been showed to positively correlate with perceived stress (38).

Generally, the relationship between psychological functions, central nervous system and immune system is mediated by different psychoneuroimmunological pathways. Neuroinflammation (39), cytokine-mediated signaling (40), and blood–brain barrier dysfunction (41) may be relevant in the development of psychiatric symptoms in systemic autoimmune disorders, including pSS. Elevated levels of pro-inflammatory cytokines such as IL-6 and TNF-α have been associated with depression, fatigue, and cognitive impairment, which are frequently reported in pSS (42). Additionally, autoantibody-mediated neural damage and small-vessel CNS vasculitis have been implicated in neuropsychiatric manifestations (43).

Focusing more on mental functions, psychosomatic symptom building, which has already been proven to be present in pSS (44), may also be a key player in seronegative cases with high anxiety levels. Thus, at higher trait anxiety scores, symptoms might occur as the result of somatization in response to physical and psychological stressors, rather than the consequence of immunobiological mechanisms. Alexithymia, which is an important feature in the psychology of pSS (45), may play an important intermediary role in psychosomatic procedures, since it is tightly linked with both trait anxiety (46) and tendencies toward somatization (47). This consequence is, however, speculative and more studies are needed to validate the intermittent role of somatization and alexithymia is symptom making in pSS.

The presence of distinct pathways leading to pSS is reinforced by a study identifying three major disease clusters (B-cell active disease and low symptom burden, high systemic disease activity, low systemic disease activity and high symptom burden) (48), suggesting different disease mechanisms, which may be the consequence of different (e.g. biological, psychological) genetical vulnerability. In addition, the result might be a consequence of a more trivial cause (e.g. methodological artifacts or confounding variables), therefore more studies are needed in this field to clarify this association.

An interesting biological observation is the importance of platelet count in predicting both autoantibodies. A plausible explanation would be that polyclonal B-cell activation depletes the bone marrow, causing thrombocytopenia in addition to other decreased blood cell counts. In addition, complex immunopathological pathways may stand in the background, like TLR7 signaling pathway and P-selectin autoantibodies, which link to thrombocytopenia is known in pSS (49–52). These possibilities are hypothetical and require further studies to verify the exact mechanisms between platelet count and autoantibodies.

State anxiety, on the other hand, was found to be an important predictor of ESSPRI. Similar associations have been revealed in the case of rheumatoid arthritis (53) and systemic lupus erythematosus (SLE) (54). Since ESSPRI is highly based on patients’ illness perception, which is- according to the presented results- influenced by state anxiety, brief psychological interventions (such as brief CBT, psychoeducation) may help to prevent or modify negative illness perception.

Differences between biological and psychological disease burden is reinforced by the finding, that IgG and RF were important negative predictors of ESSPRI. The explanation might be the difference between immunobiological and psychosomatic disease mechanisms mentioned in the case of autoantibodies and trait anxiety. ESSPRI, as a perceived disease activity marker, may reflect more sensitively on predominantly psychologically evoked symptom burden. In addition, the pathogenesis of xerostomia might be different in patients with high perceived disease activity and low serum immunological markers, compared to those with high immunological burden; anxiety induces sympathetic nerve activation, which results in dry mouth (55).

Two facets of the TCI ware chosen as important predictors for ESSPRI in this model. On one hand, fatigue is a clinical hallmark of pSS, hence this finding might seem trivial, on the other hand, however, Fatigability, as a personality trait also has a psychobiological dimension and as such, it may help understanding of the pathogenesis of fatigue and possibly other symptoms of the disease in more depth. Temperament traits (harm avoidance, prominently) influence how patients perceive and report their symptoms (56). Those with higher fatigability may be more vigilant and sensitive to bodily sensations, thereby reporting higher disease activity. Furthermore, harm avoidance was found to correlate negatively with psychological resilience (57), resulting reduced stress tolerance and increased activity of the stress axis and inflammation. Harm avoidance (alongside with its fatigability facet) is associated with serotonin levels (58), therefore, this result raises the possibility, that serotonin metabolism may be the mutual biological background for fatigue and mood symptoms of pSS.

Pure-hearted conscience (C5) facet of the Cooperativeness trait showed negative association with ESSPRI. Cooperativeness is developed through five stages of growth, which are equal to the subscales shown in the description of the scale. The fifth one (C5) can be considered as the final level of personality growth in the compartment of cooperativeness. Compassion, helpfulness, and concern for the rights of others are correlated with self-acceptance, as realized long ago (59), explaining this trait’s positive effect on perceived disease activity. Furthermore, “purehearted” acceptance of principles suggests an advanced level of moral character development in religious traditions throughout the world, emphasizing the relationship between C5 and spirituality (17). Thus, this finding is congruent with our previous work revealing positive impact of spirituality on disease activity in pSS (60). Since C5 is a character trait, which is determined by sociocultural learning, and is modifiable through experience, findings of this study may indicate a potential association between perceived severity of pSS symptoms and social and cultural influences.

Lastly, it is notable, that except for age, none of the social variables were among the most important predictors. Given the importance of social support, which has been described in pSS before (61), it would have been rational to anticipate a higher influence of social factors especially on ESSPRI. In addition, other social and demographic features, such as gender, highest level of education, or the type of settlement the patient lived in, which are well-known health determinants (62), had negligible or no effects at all. The most congruent explanation with the objective of this is that social factors have less impact on the examined aspects of disease activity. However, it is also possible that measurement limitations stand behind this lack of effect, for instance, the applied social variables do not cover appropriately the relevant social determinants of health. Future studies are needed to reveal the disease modifying strength and characteristics of social dimension. Age, however, was an important predictor of SSA and ESSPRI, showing negative and positive association with them, respectively. This finding is consistent with another study, whereas seronegative cases with mainly exocrine dysfunction and little systemic manifestations have been met more common among older pSS patients (63). ESSPRI may be higher in older age also because many hallmark symptoms – dry mouth and eyes, fatigue, and limb pain - are also common features of old age (64).

Overall, model performances differed depending on the algorithms used. Specifically, when the outcome variables were SSA and SSB, elastic net and LASSO regression consistently outperformed SVR, whereas this pattern was not observed when the outcome was ESSPRI, as model performances were much more similar. These performance differences may be attributed to several factors. SSA and SSB may have exhibited predominantly linear relationships with the features, making elastic net and LASSO regression more suitable. This was most pronounced when SSA and SSB were predicted based on biological variables only, suggesting a rather linear relationship between these variables and the outcomes. An alternative explanation is that elastic net and LASSO produced more parsimonious models with a lower risk of overfitting compared to SVR. Similarly, the linear models also slightly outperformed SVR when ESSPRI was predicted based only on psychological variables. This finding suggests that the relationship between ESSPRI and personality- and mood-related variables may also be predominantly linear, which is plausible considering that ESSPRI is a self-reported measure of disease burden. Finally, SVR outperformed the other two algorithms when ESSPRI was predicted using all three kinds of features. A plausible interpretation is that complex and potentially non-linear interactions between biological, psychological, and social variables exist, which can be captured by SVR’s kernel trick that projects data into a higher-dimensional space.

As for future clinical and research directions, the importance of both state and trait anxiety in the applied model suggests that monitoring them in pSS may contribute to the clinical work in many ways. For instance, it provides an enhanced risk stratification, allowing to presume a higher biological vulnerability at lower trait anxiety, and higher psychological vulnerability at higher state anxiety values. These correspondences also provide more targeted, individual interventions and disease management, leading toward a holistic clinical approach, potentially reducing the frequency or severity of autoimmune flares. Furthermore, psychological diagnostic tools and interventions are often less costly than long-term pharmacological treatments. STAI or other self-reported anxiety scales, for example, could be easily registered in a routine rheumatological settings. Predicting autoantibody levels through psychological assessments – based on the study results or future research - might reduce the need for extensive immunological testing and aggressive treatments, resulting in overall cost savings. As for the therapeutic processes, brief psychological interventions, such as guided self-help CBT modules, or brief psychoeducational programs—could be implemented in daily rheumatological practice without overburdening clinical workflows (e.g., by integrating 1–2 sessions or digital tools into standard care). Also, the fact that a particular form of anxiety is among the five most important predictors for SSA, SSB and ESSPRI in the statistical model draws the attention to psychological factors in the disease’s pathophysiology. Many mental health issues are known in pSS, however, their exact role in the disease mechanism is still unclear. Future studies should involve psychological factors not examined in this study, some examples mentioned in the previous paragraph. Also, the effect of the immunological treatment on mental health issues would be another possible research direction for upcoming investigations. Neurobiological investigations would be beneficial to understand the exact psychoneuroimmunological mechanism of these variables. This knowledge may open avenues for novel therapeutic targets that modulate the immune system through stress-response pathways.

## 5 Limitations and strengths

The current study has several limitations. First, in addition to the psychological variables studied, several other psychological factors may have an impact on disease activity, such as adverse childhood experience and trauma, coping mechanisms, cognitive functions etc. This stands for the clinical parameters as well. Substance use, psychiatric co-morbidities, pharmacological treatments may also play a role as confounders or effect modifiers. Second, some of the questionnaire subscales showed low levels of internal consistency and thus, the reliability of certain results is limited. Although it is important to note, that such relatively low internal consistency of the TCI subscales have been found previously in the literature, for instance, in Greek (α= 0.51-0.83) (65), Turkish (α= 0.60-0.85) (66) and Croatian (α= 0.53-0.84) (67) sample. Third, the patients were recruited in a small geographical location and thus, the generalizability to other patient samples may be impaired. Although by using nested resampling, robust models were tried to obtain, it must be admitted that external validation would be more beneficial. Future studies may consider external validation in an independent cohort to assess generalizability. Fourth and final, the sample used for the analyses was relatively low, which further limits the generalizability of the results.

Nevertheless, despite its limitations, this study also has several strengths worth highlighting. First, a complex dataset involving biological, psychological, and social variables was analyzed, allowing for the investigation of multiple aspects of disease activity in pSS. Second, to the best of our knowledge, this is the first study to train ML algorithms on such complex data in pSS, enabling direct comparisons of the effects of different types of independent variables as well as their combined impact. Third and finally, the study revealed clinically relevant relationships between variables—for example, the association between trait anxiety and SSA as well as SSB autoantibodies. Similarly important are the identified relationships between self-reported disease activity (i.e., ESSPRI) and biological variables such as IgG and RF.

## 6 Conclusions

Although much evidence is available of disease modifying biological, psychological and social health determinants in pSS, this study is the first one to analyze their importance in one model using machine learning. For the prediction of Sjögren-specific autoantibodies, biological data showed the best predictive performance, however, trait anxiety was identified as one of the most important negative predictors for both of them. State anxiety, on the contrary, was a positive important predictor of perceived disease activity, in contrast to IgG and RF, which were negative important predictors of it. These psychobiological correlations suggest that there are different disease mechanisms and symptom burden (biological and psychosomatic) in pSS. The importance of psychological factors in predicting disease activity may pave the way toward novel, more effective and sensitive diagnostic tools and therapeutic methods and better understanding of the pathomechanism of primary Sjögren’s syndrome.

## Data Availability

The raw data supporting the conclusions of this article will be made available by the authors, without undue reservation.

## References

[1] Malinow KL Molina R Gordon B Selnes OA Provost TT Alexander EL. Neuropsychiatric dysfunction in primary Sjögren’s syndrome. Ann Intern Med. (1985) 103:344–50. doi: 10.7326/0003-4819-103-3-344 2992332

[2] Patel R Shahane A. The epidemiology of Sjögren’s syndrome. Clin Epidemiol. (2014) 6:247. doi: 10.2147/CLEP.S47399 25114590 PMC4122257

[3] Miller FW. The increasing prevalence of autoimmunity and autoimmune diseases: an urgent call to action for improved understanding, diagnosis, treatment, and prevention. Curr Opin Immunol. (2023) 80:102266. doi: 10.1016/J.COI.2022.102266 36446151 PMC9918670

[4] Hernández-Molina G Leal-Alegre G Michel-Peregrina M. The meaning of anti-Ro and anti-La antibodies in primary Sjögren’s syndrome. Autoimmun Rev. (2011) 10:123–5. doi: 10.1016/J.AUTREV.2010.09.001 20833272

[5] Valtýsdóttir ST Gudbjörnsson B Lindqvist U Hällgren R Hetta J. Anxiety and depression in patients with primary Sjogren’s syndrome. J Rheumatol. (2000) 27:165–9. https://europepmc.org/article/med/10648034. 10648034

[6] Cui Y Xia L li L Zhao Q Chen S Gu Z. Anxiety and depression in primary Sjögren’s syndrome: A cross-sectional study. BMC Psychiatry. (2018) 18:131. doi: 10.1186/s12888-018-1715-x 29769121 PMC5956972

[7] Segal BM Pogatchnik B Holker E Liu H Sloan J Rhodus N. Primary Sjogren’s syndrome: Cognitive symptoms, mood, and cognitive performance. Acta Neurol Scand. (2012) 125:272–8. doi: 10.1111/j.1600-0404.2011.01530.x PMC3188671 21651503

[8] Hietaharju A Yli-Kerttula U Häkkinen V Frey H. Nervous system manifestations in Sjögren’s syndrome. Acta Neurol Scand. (1990) 81:144–52. doi: 10.1111/j.1600-0404.1990.tb00951.x 2327235

[9] Karaiskos D Mavragani CP Sinno MH Déchelotte P Zintzaras E Skopouli FN. Psychopathological and personality features in primary Sjögren’s syndrome-associations with autoantibodies to neuropeptides. Rheumatology. (2010) 49:1762–9. doi: 10.1093/rheumatology/keq158 20525741

[10] Milic V Grujic M Barisic J Marinkovic-Eric J Duisin D Cirkovic A. Personality, depression and anxiety in primary Sjogren’s syndrome – Association with sociodemographic factors and comorbidity. PloS One. (2019) 14:1–15. doi: 10.1371/journal.pone.0210466 PMC6336324 30653543

[11] Milic V Grujic M Marinkovic-Eric J Barisic J Duisin D Damjanov N. SAT0328 big-five personality in Sjogren syndrome – association with ESSPRI. Ann Rheum Dis. (2016) 75:786–6. doi: 10.1136/ANNRHEUMDIS-2016-EULAR.5637

[12] Wang K Li J Meng D Zhang Z Liu S. Machine learning based on metabolomics reveals potential targets and biomarkers for primary Sjogren’s syndrome. Front Mol Biosci. (2022) 9:913325/BIBTEX. doi: 10.3389/FMOLB.2022.913325/BIBTEX 36133908 PMC9483105

[13] Yang K Wang Q Wu L Gao QC Tang S. Development and verification of a combined diagnostic model for primary Sjögren’s syndrome by integrated bioinformatics analysis and machine learning. Sci Rep. (2023) 13:8641. doi: 10.1038/S41598-023-35864-4 37244954 PMC10224947

[14] Olivier A Hoffmann C Jousse-Joulin S Mansour A Bressollette L Clement B. Machine and deep learning approaches applied to classify Gougerot–Sjögren syndrome and jointly segment salivary glands. Bioengineering. (2023) 10:1283. doi: 10.3390/BIOENGINEERING10111283 38002406 PMC10668981

[15] Mahesh B. Machine learning algorithms - A review. Int J Sci Res (IJSR). (2020) 9:381–6. doi: 10.21275/ART20203995

[16] De Fruyt F Van De Wiele L Van Heeringen C. Cloninger’s psychobiological model of temperament and character and the five-factor model of personality. Pers Individ Dif. (2000) 29:441–52. doi: 10.1016/S0191-8869(99)00204-4

[17] Cloninger CR Svrakic DM Przybeck TR. A psychobiological model of temperament and character. Arch Gen Psychiatry. (1993) 50:975–90. doi: 10.1001/ARCHPSYC.1993.01820240059008 8250684

[18] Cloninger CR Svrakic DM Bayon C Przybeck TR. Measurement of psychopathology as variants of personality. In: Cloninger CR, editor. Personality and psychopathology. Washington DC & London, England: American Psychiatric Association (1999). p. 33–65.

[19] Rózsa S Kállai J Osváth A Bánki MC. Temperamentum és Karakter: Cloninger pszichobiológiai modellje. A Cloninger-féle temperamentum és karakter kérdőív felhasználói kézikönyve. Budapest: Medicina (2005).

[20] Beck AT Ward CH Mendelson M Mock J Erbaugh J. An inventory for measuring depression. Arch Gen Psychiatry. (1961) 4:561–71. doi: 10.1001/ARCHPSYC.1961.01710120031004 13688369

[21] Kopp M Skrabski Á Czakó L. Összehasonlító mentálhigiénés vizsgálatokhoz ajánlott módszertan. Végeken. (1990) 1–2:4–24.

[22] Rózsa S Szádóczky E Füredi J. A Beck depresszió kérdőív rövidített változatának jellemzői hazai mintán. [Psychometric properties of the Hungarian version of the shortened Beck Depression Inventory. Psychiatria Hungarica. (2001) 16:384–402.

[23] Spielberger CD Gorsuch RL Lushene R Vagg PR Jacobs GA. Manual for the State-Trait Anxiety Inventory. Palo Alto, CA: Consulting Psychologists Press (1983).

[24] Sipos K Sipos M Spielberger CD. A State-Trait Anxiety Inventory (STAI) magyar változata. In: Mérei F Szakács F, editors. Pszichodiagnosztikai vademecum I/2. Nemzeti Tankönyvkiadó, Budapest (1994). p. 123–48.

[25] Forsman L Johnson M. Dimensionality and validity of two scales measuring different aspects of self-esteem. Scand J Psychol. (1996) 37:1–15. doi: 10.1111/J.1467-9450.1996.TB00635.X

[26] Komlósi A V Rózsa S S Nagy Z Sági A Köteles F Jónás E. A vonásönbecsülés/-önértékelés kérdőíves mérésének lehetőségei. Alkalmazott Pszichológia. (2017) 17:73–108. doi: 10.17627/ALKPSZICH.2017.2.73

[27] Sherbourne CD Stewart AL. The MOS social support survey. Soc Sci Med. (1991) 32:705–14. doi: 10.1016/0277-9536(91)90150-B 2035047

[28] Moser A Stuck AE Silliman RA Ganz PA Clough-Gorr KM. The eight-item modified Medical Outcomes Study Social Support Survey: psychometric evaluation showed excellent performance. J Clin Epidemiol. (2012) 65:1107. doi: 10.1016/J.JCLINEPI.2012.04.007 22818947 PMC4119888

[29] Szentiványi-Makó H Bernáth L Szentiványi-Makó N Veszprémi B Vajda DB Kiss EC. A MOS SSS – társas támasz mérésére szolgáló kérdőív magyar változatának pszichometriai jellemzői. Alkalmazott Pszichológia. (2016) 16:145–62. doi: 10.17627/ALKPSZICH.2016.3.145

[30] Seror R Ravaud P Mariette X Bootsma H Theander E Hansen A. EULAR Sjogren’s Syndrome Patient Reported Index (ESSPRI): development of a consensus patient index for primary Sjogren’s syndrome. Ann Rheum Dis. (2011) 70:968–72. doi: 10.1136/ARD.2010.143743 21345815

[31] Pedregosa F Varoquaux G Gramfort A Michel V Thirion B Grisel O. Scikit-learn: machine learning in python. J Mach Learn Res. (2011) 12:2825–30. Available at: http://jmlr.org/papers/v12/pedregosa11a.html.

[32] Supratak A Datta G Gafson AR Nicholas R Guo Y Matthews PM. Remote monitoring in the home validates clinical gait measures for multiple sclerosis. Front Neurol. (2018) 9:561. doi: 10.3389/FNEUR.2018.00561 30057565 PMC6053510

[33] Zhao LP Bolouri H Zhao M Geraghty DE Lernmark Å. An object-oriented regression for building disease predictive models with multiallelic HLA genes. Genet Epidemiol. (2016) 40:315–32. doi: 10.1002/GEPI.21968 PMC4834870 27080919

[34] Xu WD Chen YY Wang X Su LC Huang AF. Development and external validation of a prediction model for interstitial lung disease in systemic lupus erythematosus patients: A cross-sectional study. Semin Arthritis Rheum. (2024) 69:152556. doi: 10.1016/J.SEMARTHRIT.2024.152556 39405609

[35] Matuz A van der Linden D Darnai G Csathó Á. Generalisable machine learning models trained on heart rate variability data to predict mental fatigue. Sci Rep. (2022) 12:20023. doi: 10.1038/S41598-022-24415-Y 36414673 PMC9681752

[36] Franceschini F Cavazzana I. Anti-Ro/SSA and La/SSB antibodies. Autoimmunity. (2005) 38:55–63. doi: 10.1080/08916930400022954 15804706

[37] Milin M Cornec D Chastaing M Griner V Berrouiguet S Nowak E. Sicca symptoms are associated with similar fatigue, anxiety, depression, and quality-of-life impairments in patients with and without primary Sjögren’s syndrome. Joint Bone Spine. (2016) 83:681–5. doi: 10.1016/j.jbspin.2015.10.005 26774177

[38] Bergdahl J Bergdahl M. Perceived stress in adults: prevalence and association of depression, anxiety and medication in a Swedish population. Stress Health. (2002) 18:235–41. doi: 10.1002/SMI.946

[39] Annunziata P. Neuroinflammation and Sjogren’s Syndrome. In: Mitoma H Manto M, editors. Neuroimmune Diseases. Contemporary Clinical Neuroscience. Springer, Cham (2019). p. 699–709. doi: 10.1007/978-3-030-19515-1_23

[40] Alqahtani B Daghestani M Omair MA Alhamad EH Tashkandy Y Othman N. Association of inflammatory cytokine levels with extra glandular manifestations, fatigue, and disease activity in primary Sjögren’s syndrome in Saudi patients: A cross-sectional study. Diagnostics. (2023) 13:3036. doi: 10.3390/DIAGNOSTICS13193036 37835779 PMC10572739

[41] Lauvsnes MB Tjensvoll AB Maroni SS Kvivik I Grimstad T Greve OJ. The blood–brain barrier, TWEAK, and neuropsychiatric involvement in human systemic lupus erythematosus and primary Sjögren’s syndrome. Lupus. (2018) 27:2101–11. doi: 10.1177/0961203318804895 30282561

[42] Miglianico L Cornec D Devauchelle-Pensec V Berrouiguet S Walter M Stéphan F. Inflammatory biomarkers associated with depression, anxiety, and/or fatigue in primary Sjögren’s syndrome – a systematic review. Eur J Psychiatry. (2022) 36:143–51. doi: 10.1016/J.EJPSY.2022.04.002

[43] Jaskólska M Rytlewska M Dułak NA Ulanowski M Kwarciany M Wiglusz MS. Diversity of central nervous system manifestations in Sjogren’s Disease: a case-based review. Rheumatol Int. (2025) 45:35. doi: 10.1007/S00296-024-05753-8/FIGURES/6 39836271 PMC11750892

[44] Mandl T Granberg V Apelqvist J Wollmer P Manthorpe R Jacobsson LTH. Autonomic nervous symptoms in primary Sjögren’s; syndrome. Rheumatology. (2008) 47:914–9. doi: 10.1093/RHEUMATOLOGY/KEN107 18411214

[45] van Leeuwen N Bossema ER van Middendorp H Kruize AA Bootsma H Bijlsma JWJ. Dealing with emotions when the ability to cry is hampered: emotion processing and regulation in patients with primary Sjogren’s syndrome. Clin Exp Rheumatol. (2012) 30:492–8. https://research.rug.nl/en/publications/dealing-with-emotions-when-the-ability-to-cry-is-hampered-emotion. 22512787

[46] Berthoz S Consoli S Perez-Diaz F Jouvent R. Alexithymia and anxiety: Compounded relationships? A psychometric study. Eur Psychiatry. (1999) 14:372–8. doi: 10.1016/S0924-9338(99)00233-3 10683621

[47] Mattila AK Kronholm E Jula A Salminen JK Koivisto AM Mielonen RL. Alexithymia and somatization in general population. Psychosom Med. (2008) 70:716–22. doi: 10.1097/PSY.0B013E31816FFC39 18596251

[48] Nguyen Y Nocturne G Henry J Ng WF Belkhir R Desmoulins F. Identification of distinct subgroups of Sjögren’s disease by cluster analysis based on clinical and biological manifestations: data from the cross-sectional Paris-Saclay and the prospective ASSESS cohorts. Lancet Rheumatol. (2024) 6:e216–25. doi: 10.1016/S2665-9913(23)00340-5 PMC10949202 38437852

[49] Zhang S Qu J Wang L Li M Xu D Zhao Y. Activation of toll-like receptor 7 signaling pathway in primary Sjögren’s syndrome-associated thrombocytopenia. Front Immunol. (2021) 12:637659/FULL. doi: 10.3389/FIMMU.2021.637659/FULL 33767707 PMC7986855

[50] Wu CH Li KJ Yu CL Tsai CY Hsieh SC. Sjögren’s syndrome antigen B acts as an endogenous danger molecule to induce interleukin-8 gene expression in polymorphonuclear neutrophils. PloS One. (2015) 10:e0125501. doi: 10.1371/JOURNAL.PONE.0125501 25915936 PMC4411107

[51] Bester J Pretorius E. Effects of IL-1β, IL-6 and IL-8 on erythrocytes, platelets and clot viscoelasticity. Sci Rep. (2016) 6:32188. doi: 10.1038/SREP32188 27561337 PMC4999875

[52] Hu YH Zhou PF Long GF Tian X Guo YF Pang AM. Elevated plasma P-selectin autoantibodies in primary Sjögren syndrome patients with thrombocytopenia. Med Sci Monit. (2015) 21:3690–5. doi: 10.12659/MSM.895144 PMC4668912 26613867

[53] Matcham F Ali S Irving K Hotopf M Chalder T. Are depression and anxiety associated with disease activity in rheumatoid arthritis? A prospective study. BMC Musculoskelet Disord. (2016) 17:1–9. doi: 10.1186/S12891-016-1011-1/TABLES/4 27068100 PMC4827220

[54] Nowicka-Sauer K Hajduk A Kujawska-Danecka H Banaszkiewicz D Smoleńska Czuszyńska Z. Illness perception is significantly determined by depression and anxiety in systemic lupus erythematosus. Lupus. (2018) 27:454–60. doi: 10.1177/0961203317751858 29325492

[55] Ristevska I. Xerostomia: understanding the diagnosis and the treatment of dry mouth. J Fam Med Dis Prev. (2015) 1:1–5. doi: 10.23937/2469-5793/1510008

[56] Gulec M Gulec H Oztuna F Kose S. Cloninger’s temperament and character dimension of personality in patients with asthma. Int J Psychiatry Med. (2010) 40:273–87. doi: 10.2190/PM.40.3.D 21166338

[57] Acar Sivri G Ezgi Ünal F Güleç H. Resilience and personality in psychiatric inpatients. Psychiatry Clin Psychopharmacol. (2019) 29:650–5. doi: 10.1080/24750573.2018.1540199

[58] Szekely A Ronai Z Nemoda Z Kolmann G Gervai J Sasvari-Szekely M. Human personality dimensions of persistence and harm avoidance associated with DRD4 and 5-HTTLPR polymorphisms. Am J Med Genet B Neuropsychiatr Genet. (2004) 126B:106–10. doi: 10.1002/AJMG.B.20134 15048658

[59] Berger EM. The relation between expressed acceptance of self and expressed acceptance of others. J Abnorm Psychol. (1952) 47:778–82. doi: 10.1037/H0061311 12999407

[60] Módis LV Aradi Z Horváth IF Pikó P Papp G Osváth M. Spirituality is associated with immune parameters and disease activity in primary Sjögren’s syndrome: a cross-sectional study. Sci Rep. (2024) 14:12473. doi: 10.1038/S41598-024-62801-W 38816520 PMC11139944

[61] Karaiskos D Mavragani CP Makaroni S Zinzaras E Voulgarelis M Rabavilas A. Stress, coping strategies and social support in patients with primary Sjögren’s syndrome prior to disease onset: a retrospective case–control study. Ann Rheum Dis. (2009) 68:40–6. doi: 10.1136/ARD.2007.084152 18276740

[62] Braveman P Gottlieb L. The social determinants of health: it’s time to consider the causes of the causes. Public Health Rep. (2014) 129:19. doi: 10.1177/00333549141291S206 PMC3863696 24385661

[63] Lan J Deng C Huang H Rao P Chen Y Shi Y. Seronegative primary Sjögren’s syndrome, a distinct subtype of primary Sjögren’s syndrome in Chinese patients. BMC Rheumatol. (2024) 8:15. doi: 10.1186/S41927-024-00384-9 38627838 PMC11020423

[64] Moerman RV Bootsma H Kroese FGM Vissink A. Sjögren’s syndrome in older patients: aetiology, diagnosis and management. Drugs Aging. (2013) 30:137–53. doi: 10.1007/S40266-013-0050-7 23341116

[65] Fountoulakis KN Rozsa S Siamouli M Moutou K Pantoula E Cloninger CR. Standardization and normative data of the Greek version of the temperament and character inventory (TCI). Ann Gen Psychiatry. (2015) 14:28. doi: 10.1186/S12991-015-0067-X 26396587 PMC4578673

[66] Arkar H Sorias O Tunca Z Safak C Alkin T Binnur Akdede B. Factorial structure, validity, and reliability of the Turkish temperament and character inventory. Turk Psikiyatri Derg. (2005) 16:190–204. 16180152

[67] Jaksic N Aukst-Margetic B Rózsa S Brajkovic L Jovanovic N Vuksan-Cusa B. Psychometric properties and factor structure of the Temperament and Character Inventory-Revised (TCI-R) in a Croatian psychiatric outpatient sample. Compr Psychiatry. (2015) 57:177–86. doi: 10.1016/J.COMPPSYCH.2014.10.016 25464839

